# Treadmill exercise protects against methylmercury neurotoxicity by increasing BDNF in the mouse brain

**DOI:** 10.1265/ehpm.25-00360

**Published:** 2025-12-04

**Authors:** Masatake Fujimura

**Affiliations:** Department of International Affairs and Research, National Institute for Minamata Disease, Minamata, Japan

**Keywords:** Methylmercury, Treadmill exercise, BDNF, Cerebral cortex

## Abstract

**Background:**

Methylmercury (MeHg) causes damage specifically in cerebrocortical neurons, but not in hippocampal neurons. In our previous studies using cultured neurons, we found that brain-derived neurotrophic factor (BDNF), which is prominently present in hippocampal neurons, plays a key role in resistance to MeHg neurotoxicity. Our findings, combined with recent findings that moderate exercise increases BDNF in the brain, led us to hypothesize that moderate exercise protects against MeHg-induced neurotoxicity by inducing BDNF expression.

**Methods:**

C57 black 6NJcl (C57BL/6NJcl) male mice were used to evaluate the effects of treadmill exercise (a moderate exercise) on the neurotoxicity of MeHg exposure at 1.5 mg/kg/day. The effects of treadmill exercise on MeHg neurotoxicity were evaluated through neurobehavioral, neuropathological, and biochemical analyses using brain tissue, blood, and muscle tissue.

**Results:**

Treadmill exercise had a significant inhibitory effect on the neurological symptoms associated with apoptotic neuronal death and subsequent cerebrocortical neuron loss induced by MeHg exposure. In the cerebral cortex, treadmill exercise significantly increased BDNF levels and activated the neuroprotective-related BDNF-tropomyosin receptor kinase (Trk) B and p44/42 mitogen-activated protein kinase (MAPK) pathways along with significantly suppressing the neuronal cell death-associated p38 MAPK pathway. Furthermore, treadmill exercise significantly increased fibronectin type III domain containing 5 (FNDC5) expression in the muscle tissue and elevated ed the concentration of its metabolite, irisin, in the blood.

**Conclusions:**

These results suggest that treadmill exercise increases BDNF in the brain and suppresses neurotoxic pathways, ultimately protecting against MeHg neurotoxicity. Moreover, the increase of BDNF in the brain may be attributed to the exercise-induced increased expression of FNDC5 in muscle tissue from where it is released into the blood as irisin and finally transferred into the brain and promoted BDNF production.

## Background

Minamata disease, also referred to as methylmercury (MeHg) poisoning, is a neurological disease that was discovered to be caused by the runoff of MeHg from a chemical plant and subsequent ingestion through contaminated seafood in Japan [1–3]. Currently, MeHg runoff from chemical plants is almost nonexistent, however, mercury contamination from gold mining and subsequent MeHg contamination in the food chain remain a noteworthy concern globally, including in Southeast Asia, Latin America [4]. MeHg toxicosis leads to neuronal damage, ultimately causing neurological dysfunction. After nerve cell damage occurs, regenerating those nerve cells is nearly impossible, with recovery from nerve dysfunction also being exceedingly difficult. In light of these problems, preventive medical treatment, which is provided before nerve dysfunction occurs, is a necessary countermeasure against MeHg intoxication.

MeHg exposure in adulthood is mainly characterized by neuronal degeneration in the limbic cortex [5–7], causing sensory and motor dysfunction [2, 8, 9]. In contrast, adult exposure to MeHg is not toxic to hippocampal neurons in the same limbic system that is highly vulnerable to various insults such as ischemia, hypoglycemia, and external stimuli [10–14]. This selective neuronal toxicity of MeHg has been reproduced in experimental animal models using rodents [15, 16] and primary neuronal cell cultures [17], revealing that a type of neurotrophic factor (NTF), brain-derived neurotrophic factor (BDNF), is specifically present in the MeHg neurotoxicity-resistant hippocampal neurons, but not in the cortical neurons vulnerable to MeHg neurotoxicity. Furthermore, experiments using primary neuronal cell cultures with the loss or addition of BDNF highlighted that BDNF is a crucial factor in MeHg tolerance and that the subsequent activation of the BDNF-tropomyosin receptor kinase (Trk) B and p44/42 mitogen-activated protein kinase (MAPK) pathways is involved in the neuroprotective action of BDNF [18].

Prior studies have also demonstrated that BDNF, a widely present NTF in the central nervous system, binds to the TrkB receptor and activates intracellular signaling, thereby contributing to cell survival, growth, and synaptic plasticity [19, 20]. In addition, exercise has been found to enhance BDNF expression in the brain, with Neeper et al. being the first researchers to report this finding [21]. Subsequent studies have shown that moderate exercise improves BDNF expression in various brain regions such as the cerebral cortex, hippocampus, striatum, and cerebellum as well as the spinal cord [22–25]. Other researchers have suggested that moderate exercise heightens the expression of NTFs, including BDNF, in diverse regions, which may contribute to inducing plasticity and maintaining neural function, such as neurogenesis, enhancement of circuit function, and neuroprotection [26–29]. Moreover, basic studies in rodent models often use aerobic exercise on treadmills or spinning wheels to examine the effects of exercise, whereas studies involving humans have shown that blood BDNF levels increase transiently after aerobic exercises, such as those on bicycle ergometers [30].

The neural activity-dependent increase in BDNF expression is a well-established concept [31]. Therefore, the neural activity that occurs with physical exercise may be the mechanism of increased BDNF expression during exercise [32]. In addition to these mechanisms in the brain, several factors and metabolites from peripheral tissues have recently been found to influence BDNF expression in the brain. Among them, myokines such as fibronectin type III domain-containing 5 (FNDC5), which is converted to irisin in the bloodstream, are produced in muscle, and act directly (or indirectly) via the blood to the brain have been suggested to increase BDNF expression [33, 34]. Interestingly, administering plasma from exercised mice to non-exercised mice has been observed to induce effects such as BDNF expression and neurogenesis in the non-exercised mice [35, 36], suggesting that the skeletal muscle, circulatory system, and brain play critical role in the effects of exercise systems. Thus, recent studies have gradually shown that exercise augments BDNF expression via diverse systems in vivo.

Based on the above findings, we hypothesized that moderate exercise may suppress MeHg-induced neurotoxicity by promoting an increase in BDNF in the brain and tested this hypothesis by conducting an experimental animal study using mice. In addition, we performed detailed analyses of the molecular mechanisms underlying this exercise-induced effect in the brain, blood, and muscle.

## Materials and methods

### Experimental animals and ethics statements

Thirty-two 7-week-old C57 black 6NJcl (C57BL/6NJcl) male mice (body weight, 19–22 g) were purchased from CLEA Japan (Tokyo, Japan) to conduct the animal experiments. All mice were housed in cages (four per cage) under a 12/12-h light/dark cycle with free access to food and water at a temperature of 24 °C. All animal experiments were performed in accordance with the “Guide for the Care and Use of Laboratory Animals” issued by the National Institute for Minamata Disease.

### Administration of MeHg

After 1 week of acclimation, mice were randomly divided into four groups of eight animals each (vehicle + no exercise, MeHg + no exercise, vehicle + exercise, MeHg + exercise). MeHg was purchased from Tokyo Chemical Industry Co., Ltd. (Tokyo, Japan), and the MeHg solution was prepared to a concentration of 30 ppm with MeHg-glutathione (1:1) complex, according to our previous paper [37]. This MeHg solution was administered in drinking water for 6 weeks. A daily MeHg intake of 1.5 mg/kg was controlled at the cage level by adjusting the amount of drinking water provided [38], whereas the vehicle group was administered a MeHg-free glutathione solution.

### Treadmill exercise protocol

In the exercise group, a mouse-specific treadmill device without electrical stimulation (TMS-M4SL; MELQUEST, Toyama, Japan) [39, 40] was used to make the mice perform daily treadmill exercise for 30 min over a 6-week period. The exercise speed was 10 m/min (incline: 0%), which is considered moderate exercise [41–44] and is almost equivalent to jogging in humans. The mice in the non-exercise group were not made to exercise on the treadmill.

### Assessment of neurological symptoms and body weight

Abnormal hind limb extension, a specific sign of neurological dysfunction in MeHg-intoxicated rodent models, was evaluated once a week during the 6 weeks of MeHg administration to determine neurological symptoms [45–47]. In this assessment, mice were gently removed from their cages and hung upside down by their tails for a few seconds. Hind limb extension was evaluated using the following criteria: no hind limb extension at all and fully crossed = 3, no hindlimb extension at all = 2, incomplete extension = 1, and normal extension = 0. Body weight was also measured once a week during the 6 weeks of MeHg administration.

### Evaluation of neuropathological changes

Neuropathological changes were investigated by performing pathological staining, including immunohistochemistry, of the sections. In this process, brain tissues were extracted from the mice after placing them under deep anesthesia with isoflurane (FUJIFILM Wako Pure Chemicals, Osaka, Japan). Next, the brain tissue samples were immersed in formalin, embedded in paraffin, and then prepared into 5-µm coronal sections using a microtome (Leica RM2245; Leica Biosystems, Nussloch, Germany) [15]. The sections were then stained using antigen-specific antibodies [48] for neuronal nuclei (NeuN) (Chemicon, Temecula, CA, USA), ionized calcium-binding adaptor molecule 1 (Iba1) (FUJIFILM Wako Pure Chemicals), glial fibrillary acidic protein (GFAP) (Dako Corporation, Carpinteria, CA, USA), and BDNF (GenTex, Zeeland, MI, USA). The nuclei were counterstained with hematoxylin (Dako Corporation). Apoptotic cell death was measured by deoxynucleotidyl transferase-mediated dUTP nick-end labeling (TUNEL) staining (In Situ Cell Death Detection Kit, TMR red; Roche, Basel, Switzerland) [47], along with nuclei counterstaining using Vibrance Antifade Mounting Medium containing 4′,6-diamidino-2-phenylindole (DAPI) (Vector Laboratories, Burlingame, CA, USA).

### Measurement of protein expression levels in the brain and muscle

Western blotting analysis was conducted to measure protein expression in the brain (i.e., in the cerebral cortex where neurological lesions were observed) and muscle (i.e., in the extensor digitorum longus muscle that plays an important role in treadmill exercise) [49]. After MeHg administration was completed, the cerebral cortex and extensor digitorum longus muscle were separated from the brain and lower limb muscles, respectively, of the mice after placing them under deep anesthesia with isoflurane. Once the tissue was stored at −80 °C, samples were prepared for western blotting analysis [15]. In this study, western blotting analysis was performed using the following antibodies against each specific antigen: nerve growth factor (NGF), neurotrophin 3 (NT3), NT4, TrkA, phospho-TrkA (Tyr490), TrkB, phospho-TrkB (Tyr-516), TrkC, phospho-TrkC (Tyr820), p44/42 MAPK, phospho-p44/42 MAPK (Thr202/Tyr204), p38 MAPK, phospho-p38 MAPK (Thr180/Tyr182), β-actin (Cell Signaling Technology, Danvers, MA, USA), BDNF (Sigma-Aldrich, St. Louis, MO, USA), and FNDC5 (Novus Biologicals, Littleton, CO, USA). The expression levels of each protein were corrected for β-actin or the respective total protein level.

### Measurement of protein expression levels in the plasma

Protein concentrations in the plasma were evaluated by enzyme-linked immunosorbent assay. After the completion of MeHg administration, a heparinized syringe was used to draw blood from the heart of the mice that were placed under deep anesthesia with isoflurane. Plasma was immediately separated from the blood by centrifugation and used for protein analysis. Plasma irisin and BDNF levels were measured using the Irisin EIA/ELISA kit (Phoenix Pharmaceuticals Inc., Burlingame, CA, USA) and Mature BDNF ELISA Kit Wako (FUJIFILM Wako Pure Chemicals).

### Measurement of mercury levels in the blood and tissue

After the completion of MeHg administration, blood and tissue samples were collected, and their total mercury concentrations were measured using heating vaporization atomic absorption spectrometry using a mercury analyzer (MA2000; Nippon Instruments, Tokyo, Japan) [50, 51].

### Statistical analysis

**Fig. 1:**
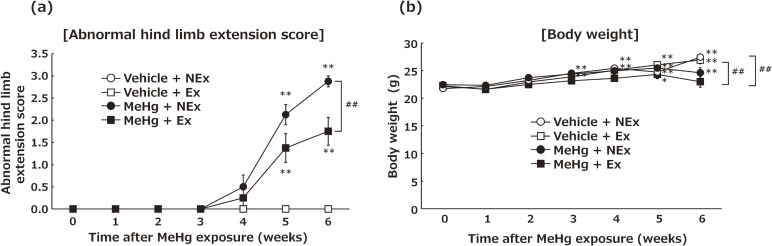
Effects of MeHg exposure and treadmill exercise on hind limb extension and body weight. (a) Hind limb extension. (b) Body weight. Values are expressed as mean ± SEM (8 mice per group). One-way analysis of variance using Wilcoxon signed-rank test in Fig. 1a and Dunnett’s method in Fig. 1b confirmed significant differences in baseline levels within each group. One-way analysis of variance using Tukey’s method confirmed significant differences in intergroup comparisons. Abbreviations: Ex, exercise; NEx, no exercise.

Baseline levels within each group were compared by conducting one-way analysis of variance using Wilcoxon signed-rank test in Fig. 1a and Dunnett’s method in Fig. 1b, whereas intergroup comparisons were performed through one-way analysis of variance using Tukey’s method. Student’s t-test was used for significance testing between two groups. Data were expressed as mean ± standard error of the mean (SEM), with *p* < 0.05 considered statistically significant.

## Results

### Treadmill exercise

Preliminary investigations were conducted regarding the conditions for treadmill exercise. The speed of 10 m/min used in this study represents the maximum feasible speed; it was established that higher speeds cause animals to fall from the apparatus.

### Effects of MeHg exposure and treadmill exercise on hind limb extension and body weight

In mice without treadmill exercise but continuous exposure to 30 ppm of MeHg, abnormal hind limb extension was observed from the 4 week of exposure. In contrast, mice exposed to MeHg during treadmill exercise exhibited significantly fewer abnormalities in hind limb extension than those in the non-exercise group with MeHg exposure (Fig. 1a). No abnormalities in hind limb extension were observed in the groups not exposed to MeHg and with or without treadmill exercise. In terms of body weight changes, most experimental groups showed significant weight gain from 4 or 5 week of exposure, with only the MeHg + exercise group showing no significant weight gain (Fig. 1b). At week 6, the MeHg + exercise group weighed significantly less than the unexposed groups, although the difference was only approximately 10%.

### Effects of MeHg exposure and treadmill exercise on neuropathological changes in the cerebral cortex

**Fig. 2:**
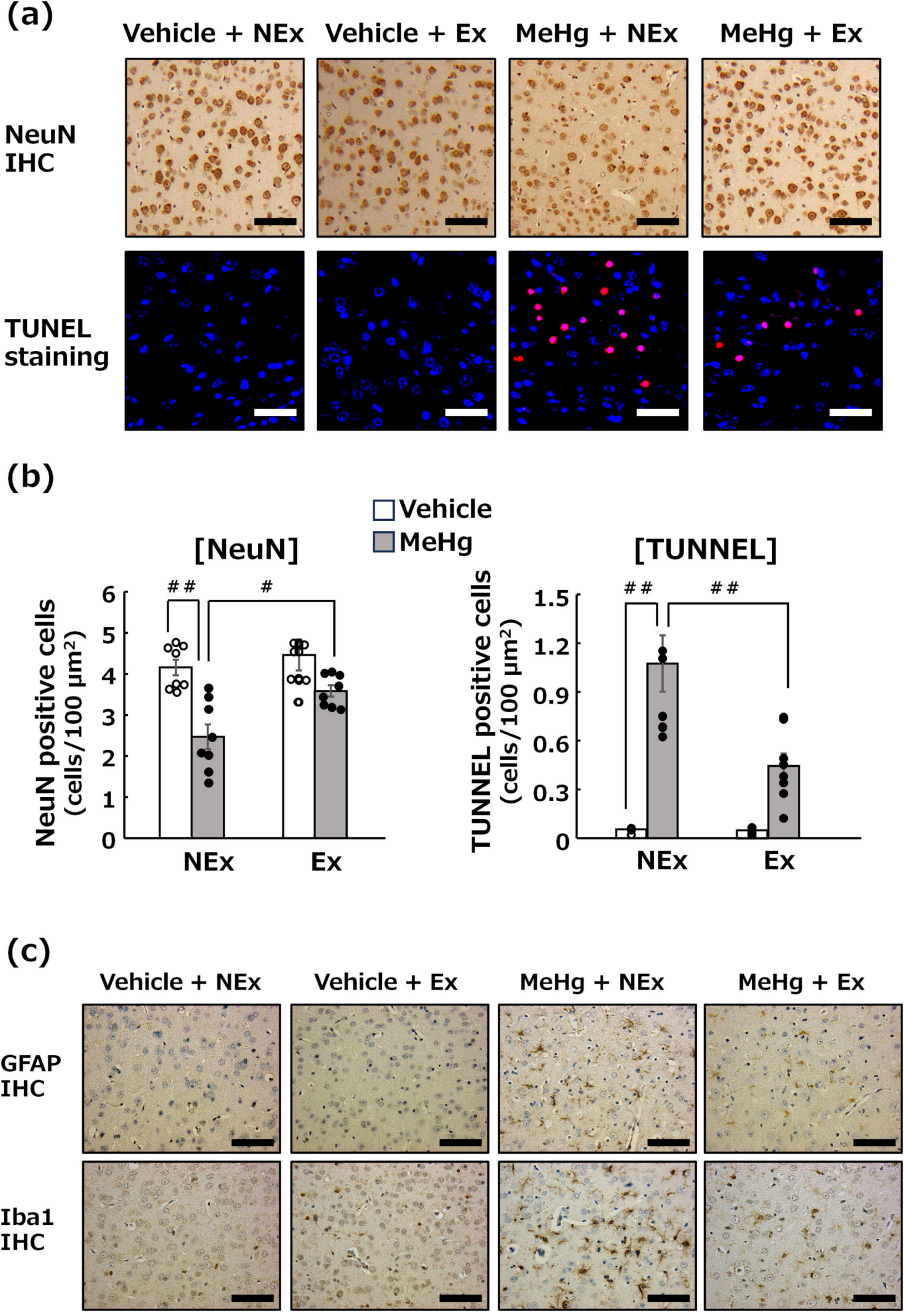
Effects of MeHg exposure and treadmill exercise on neuropathological changes. (a) Upper panels show NeuN immunostaining (brown nuclei) in the cerebral cortex, which stains explicitly neuronal nuclei. Lower panels display TUNEL-positive cells (red nuclei) in the cerebral cortex, an indicator of apoptotic neuronal cell death. Bar = 25 µm. (b) Statistical comparison of the number of NeuN- and TUNEL-positive neurons. Values are expressed as mean ± SEM (8 mice per group). One-way analysis of variance using Tukey’s method confirmed significant differences in intergroup comparisons (^#^p < 0.05, ^##^p < 0.01). (c) Immunostaining for GFAP or Iba1-positive cells reveals activated astroglia or activated microglia (brawn cells), respectively, which occur during neuronal injury. Bar = 25 µm. Abbreviations: Ex, exercise; NEx, no exercise.

Immunohistochemical staining for NeuN, a protein specifically expressed in neurons, revealed that MeHg exposure specifically reduced neurons in the deep cerebral cortex (Fig. 2a, upper panel). Moreover, TUNEL-positive cells were observed in the regions where neuronal reduction occurred, confirming that apoptotic neuronal cell death was associated with MeHg-induced neuronal injury (Fig. 2a, lower panel). Treadmill exercise had a significant inhibitory effect on MeHg exposure-induced reduction in neurons and increase in TUNEL-positive cells (Fig. 2a, b). Furthermore, treadmill exercise suppressed the expressions of GFAP-positive activated astrocytes and Iba1-positive activated microglia that had increased due to MeHg exposure (Fig. 2c).

### Effects of treadmill exercise on NTF expression in the brain

**Fig. 3:**
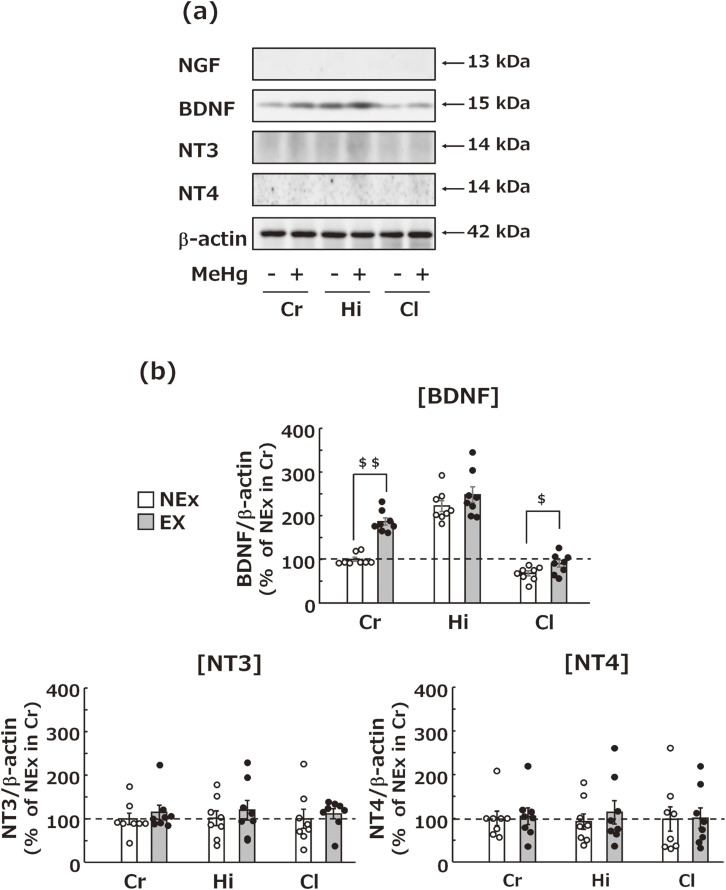
Effects of treadmill exercise on NTFs in various brain tissues. (a) Representative images of western blotting for measuring protein expression levels of NTFs (NGF, BDNF, NT3, and NT4). (b) Statistical comparison of the protein expression levels of NTs detected by western blot. Values are expressed as mean ± SEM (8 mice per group). Student’s t-test confirmed significant differences between the two designated groups (^$^p < 0.05, ^$$^p < 0.01). Abbreviations: Ex, exercise; NEx, no exercise; Cr, cerebral cortex; Hi, hippocampus; Cl, cerebellum.

We examined changes in NTFs in the brain induced by treadmill exercise and found that treadmill exercise did not affect NT3 and NT4 expression but significantly increased BDNF expression in the cerebral cortex and cerebellum (Fig. 3a). In particular, treadmill exercise increased the cerebrocortical expression of BDNF to nearly its expression levels in the hippocampus of mice without exercise (Fig. 3b). The expression levels of NGF could not be detected.

### Effects of MeHg exposure and treadmill exercise on the expression of NTFs and activation of their receptors in the cerebral cortex

**Fig. 4:**
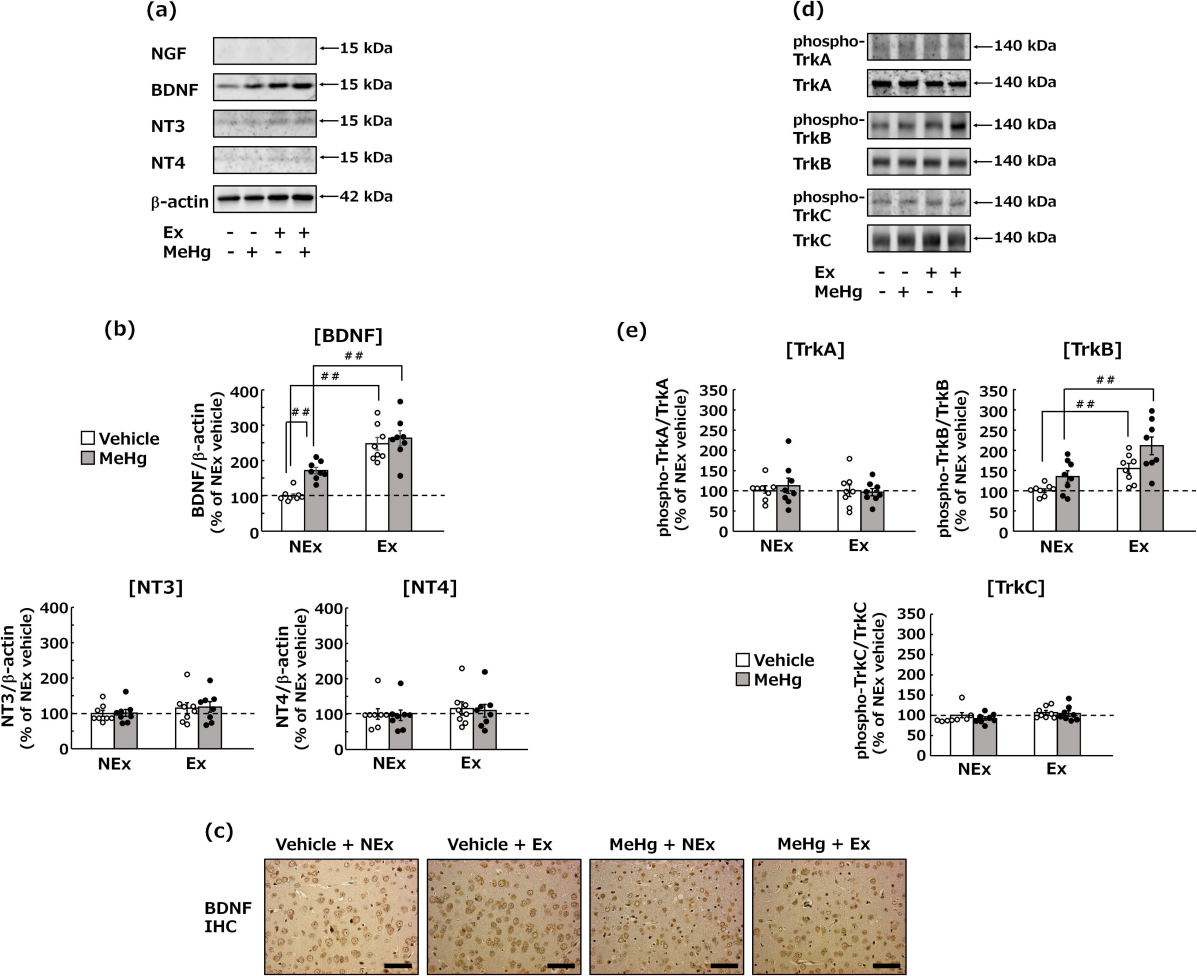
Effects of MeHg exposure and treadmill exercise on NTFs and their receptors. (a) Representative images of western blotting for estimating the protein expression levels of NTFs (NGF, BDNF, NT3, and NT4) in the cerebral cortex. (b) Statistical comparison of the protein expression levels of NTs determined by western blot. Values are expressed as mean ± SEM (8 mice per group). One-way analysis of variance using Tukey’s method confirmed significant differences in intergroup comparisons (^#^p < 0.05, ^##^p < 0.01). (c) Immunostaining for BDNF-positive cells (brown nuclei) in the cerebral cortex. Bar = 25 µm. (d) Representative images of western blotting for evaluating the activation of the Trk pathways in the cerebral cortex. (e) Statistical analysis of the activation levels of the Trk pathway obtained by western blot. Values are expressed as mean ± SEM (8 mice per group). One-way analysis of variance using Tukey’s method confirmed significant differences in intergroup comparisons (^#^p < 0.05, ^##^p < 0.01). Abbreviations: Ex, exercise; NEx: no exercise.

Treadmill exercise significantly increased BDNF levels in the cerebral cortex. MeHg exposure itself also resulted in increased BDNF levels in the cerebral cortex in the non-exercise group, but not in the exercise groups (Fig. 4a, b). In contrast, the expression of NT3 and NT4 was not affected by treadmill exercise or MeHg exposure. In addition, NGF could not be detected. The treadmill exercise-induced increase in BDNF was also confirmed by immunohistochemical staining of the nuclei of cerebrocortical neurons (Fig. 4c). Furthermore, the activation of the TrkB pathway (a BDNF and NT4 receptor) was significantly promoted in accordance with the increased BDNF levels (Fig. 4d, e). Conversely, the activation of the TrkA pathway (an NGF receptor) and the TrkC pathway (an NT3 receptor) [19] was not observed after MeHg exposure or treadmill exercise.

### Effects of MeHg exposure and treadmill exercise on kinases associated with neurotoxicity and neuroprotection in the cerebral cortex

**Fig. 5:**
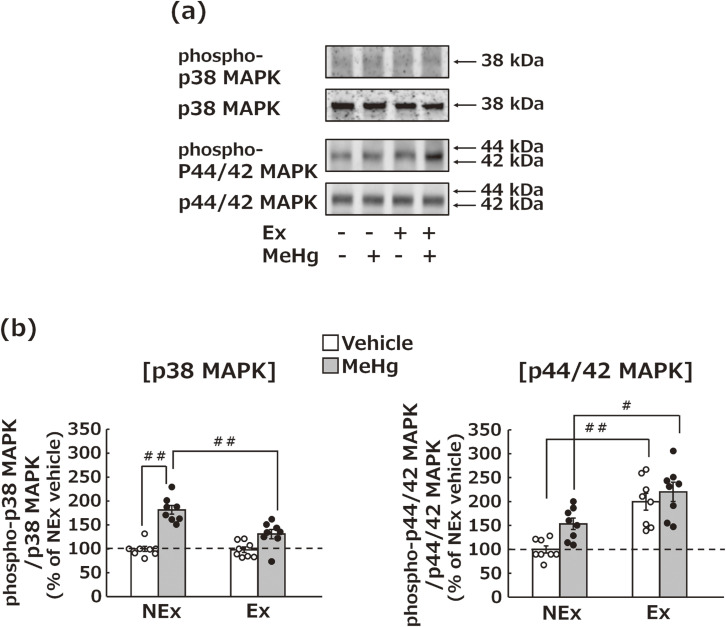
Effects of MeHg exposure and treadmill exercise on kinases associated with neurotoxicity and neuroprotection. (a) Representative images of western blotting for assessing kinase activation in the cerebral cortex. (b) Statistical comparison of the kinase activation levels detected by western blot. Values are expressed as mean ± SEM (8 mice per group). One-way analysis of variance using Tukey’s method confirmed significant differences in intergroup comparisons (^#^p < 0.05, ^##^p < 0.01). Abbreviations: Ex, exercise; NEx, no exercise.

MeHg exposure activated the p38 MAPK pathway, a type of neurotoxic kinase [52], whereas treadmill exercise significantly suppressed its activation in the cerebral cortex (Fig. 5a, b). In contrast, the p44/42 MAPK pathway, a potential neuroprotective kinase [18], was not affected by MeHg exposure, but its activation was significantly increased by treadmill exercise in the cerebral cortex (Fig. 5a, b).

### Effects of MeHg exposure and treadmill exercise on FNDC5 expression in muscle

**Fig. 6:**
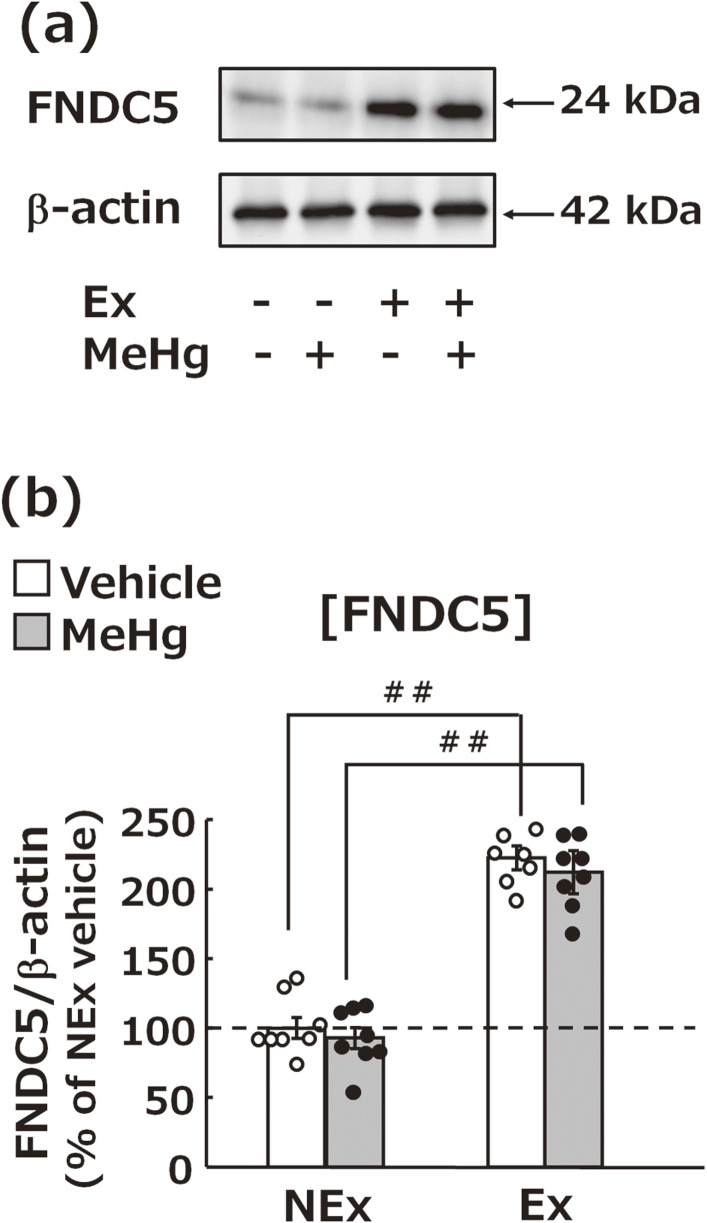
Effects of MeHg exposure and treadmill exercise on FNDC5 expression. (a) Representative images of western blotting for determining the protein expression levels of FNDC5 in the lower limb muscle tissue. (b) Statistical comparison of FNDC5 expression levels estimated by western blot. Values are expressed as mean ± SEM (8 mice per group). One-way analysis of variance using Tukey’s method confirmed significant differences in intergroup comparisons (^#^p < 0.05, ^##^p < 0.01). Abbreviations: Ex, exercise; NEx, no exercise.

FNDC5 expression in the extensor digitorum longus muscle was not affected by MeHg exposure but was significantly increased by treadmill exercise (Fig. 6a, b).

### Effects of MeHg exposure and treadmill exercise on plasma irisin and BDNF concentrations

**Fig. 7:**
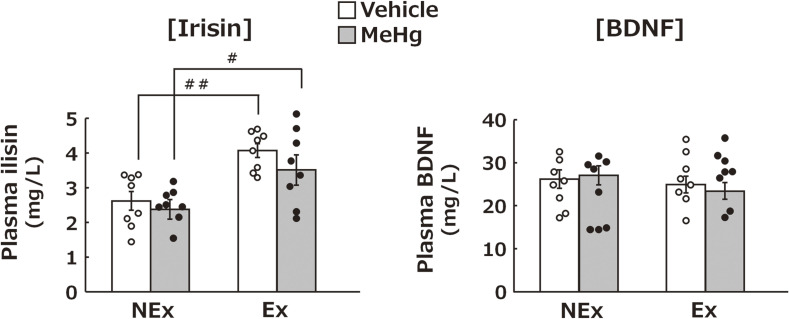
Effects of MeHg exposure and treadmill exercise on irisin and BDNF levels in the plasma. Statistical comparison of the plasma expression levels of irisin and BDNF obtained. Values are expressed as mean ± SEM (8 mice per group). One-way analysis of variance using Tukey’s method confirmed significant differences in intergroup comparisons (^#^p < 0.05, ^##^p < 0.01). Abbreviations: Ex, exercise; NEx, no exercise.

Although blood irisin levels were not affected by MeHg exposure, they were significantly increased by treadmill exercise. Plasma BDNF levels were not affected by MeHg exposure or treadmill exercise (Fig. 7).

### Mercury concentration in blood and various tissues

Similar mercury concentrations were detected in the cerebral cortex, hippocampus, and cerebellum of the mice with MeHg exposure; however, neurological lesions were only found in the cerebral cortex, but not in the hippocampus and cerebellum. Mercury concentrations in the varied brain tissues and muscle and blood were not affected by treadmill exercise (Table 1).

**Table 1:** Mercury concentration in the blood, muscle, and various brain tissues.

	**Whole blood** **(ppm)**	**Cerebral cortex** **(ppm)**	**Hippocampus** **(ppm)**	**Cerebellum** **(ppm)**	**Extensor digitorum** **longus muscle (ppm)**
Vehicle + Nex	<0.2	<0.4	<0.4	<0.4	<0.4
MeHg + Nex	17.4 ± 1.2	17.7 ± 0.4	18.1 ± 0.6	15.1 ± 0.3	9.8 ± 0.8
Vehicle + Ex	<0.2	<0.4	<0.4	<0.4	<0.4
MeHg + Ex	16.9 ± 3.4	17.4 ± 2.4	16.5 ± 2.1	14.2 ± 2.3	9.7 ± 1.6

Values are expressed as mean ± SEM (8 mice per group).

## Discussion

Exercise is widely recognized for its positive impact on health. Various guidelines recommend that the general adult population should engage in at least 150 min of moderate aerobic exercise per week to improve their overall health [53]. Moreover, recent research has revealed that exercise is not only beneficial for the body but also has varied effects on the brain and nervous system, including improving cognitive function, suppressing aging-associated degenerative changes and neurodegenerative diseases, and helping recovery from neurological disorders [54]. However, whether exercise contributes to health protection when the human body is exposed to toxic substances remains unclear. In this study, we conducted animal studies using mice to examine the effects of exercise on MeHg neurotoxicity and found that moderate exercise suppressed MeHg neurotoxicity.

The first step in this study was to determine how to apply moderate exercise to experimental animals. The average height of an adult human male is estimated to be 172.9 cm [55]. In the case of our experimental mice, the height of the male mice was approximately 13 cm, excluding their tails. The 10 m/min treadmill exercise performed by the mice in this study was converted to a speed of 133 m/min or 7.98 km/h in adult human males based on the height of the mice. Considering the above conversions, the treadmill exercise performed by the mice in this study was appropriate as a moderate exercise, comparable to jogging in humans. Mice that performed this treadmill exercise demonstrated significant suppression of the neurological symptoms caused by MeHg exposure (Fig. 1a). In this experiment, there was concern regarding the potential effects of MeHg exposure-related neurological dysfunction and weight loss (Fig. 1b) on treadmill exercise. However, all mice were able to perform the treadmill exercise despite hind limb extension dysfunction. All these findings support the validity of this experimental system when examining the effects of treadmill exercise on MeHg neurotoxicity.

The benefits of treadmill exercise in this study of MeHg exposure-induced neurological dysfunction were supported by histopathological analyses (Fig. 2). In studies using mice as experimental animals, MeHg exposure-induced neurological lesions have been shown to occur as neuronal loss and the appearance of apoptotic cells in the deep cerebral cortex, accompanied by glial cell activation [56, 57]. In the present study, treadmill exercise significantly suppressed above-mentioned neurological lesions, suggesting that treadmill exercise impedes neuronal dysfunction via its inhibitory action on neuronal lesions (Fig. 2).

Subsequently, the molecular mechanism of the positive effects of treadmill exercise was examined. For this purpose, we evaluated the expression levels of NTFs, including BDNF, in various regions of the brain. The hippocampus, which did not exhibit neuropathic lesions caused by MeHg exposure, was found to have high expression levels of BDNF. Conversely, the cerebral cortex, which was shown to have MeHg exposure-induced neuropathic lesions, was detected to have low BDNF expression (Fig. 3a). Moreover, treadmill exercise did not significantly increase BDNF in the hippocampus, but it did increase BDNF in the cortex to levels similar to those in the hippocampus of mice without treadmill exercise (Fig. 3b). These results indicate that treadmill exercise can inhibit MeHg-induced neuropathological changes via increased BDNF in the cortex. In mice, MeHg exposure does not induce neurological lesions in the cerebellum [58]. In the present study, BDNF levels in the mouse cerebellum were revealed to be lower than those in the cerebral cortex and hippocampus, and did not increase to the same level as in the hippocampus of mice that did not perform treadmill exercise after treadmill exercise. Based on the above results, defense mechanisms other than BDNF may protect the cerebellum of mice from MeHg toxicity.

We further examined the alterations in NTFs and their receptors in the cerebral cortex during MeHg exposure. BDNF showed an approximately 2-fold significant increase in response to MeHg exposure and the absence of treadmill exercise, whereas a significant 3-fold increase in BDNF was associated with by treadmill exercise (Fig. 4a–c). Furthermore, the increase in BDNF was accompanied by the specific activation of its receptor, TrkB (Fig. 4d, e), indicating that the BDNF-TrkB pathway in the cerebral cortex is activated by treadmill exercise and suppresses MeHg-induced neurotoxicity. Given that previous studies have suggested that increased BDNF in cultured neurons affects the kinase pathway involved in MeHg exposure-induced neuronal cell death in addition to the TrkB pathway [18], the kinase pathway was also examined in this animal study. This study found that MeHg exposure activated the p38 MAPK pathway, a neuronal death pathway [52, 59], but treadmill exercise significantly suppressed the activation of this pathway. In our previous research using cultured neurons, SB203580, a specific inhibitor of p38 MAPK, significantly suppressed MeHg-induced neuronal cell death [52]. This finding suggests that the suppression of p38 MAPK activity by treadmill exercise plays a crucial role in protecting against MeHg neurotoxicity. In contrast, MeHg exposure had no such effect on the p44/42 MAPK pathway, which was previously thought to be involved in the inhibition of neuronal cell death [60, 61], whereas treadmill exercise led to a significant activation of this pathway (Fig. 5). Considering all these results, BDNF may modulate MeHg exposure-induced neurotoxicity by regulating the kinase pathway involved in neuronal cell death, even at the animal experimental level.

Factors that may be involved in the increased BDNF levels in the brain were examined in terms of protein expression in the muscle and blood. In the muscle analysis, the extensor digitorum longus muscle was used. This lower limb muscle includes both fast and slow-twitch fibers required for moderate exercise such as treadmill exercise [62]. Treadmill exercise increased the levels of FNDC5 in muscles and irisin in the blood, which have been earlier implicated in increasing BDNF in the brain [33, 34] (Fig. 6, 7). Considering that FNDC5 in muscles is a precursor of irisin in blood, treadmill exercise may have increased irisin in the blood by elevating FNDC5 concentration in muscles. These findings suggest that the treadmill exercise increased FNDC5 in the muscles tissue from where it is released into the blood as irisin and finally transferred into the brain and promoted BDNF production. In contrast, blood BDNF levels did not change after 6 weeks of exercise, suggesting that blood BDNF levels do not affect the brain BDNF levels (Fig. 7). Furthermore, recent studies have reported that irisin itself possesses antioxidant [63, 65], anti-inflammatory [64, 65], mitochondrial protective [63, 66], and signaling effects [65]. Therefore, it is important to note that the suppression of exercise-induced MeHg neurotoxicity may involve the inhibitory effects of irisin itself, independent of BDNF expression enhancement. Elucidating the detailed relationship between FNDC5-ilisin-BDNF pathway and MeHg neurotoxicity remains a subject for future investigation.

In this study, it was necessary to measure the effects of treadmill exercise on mercury concentrations in each tissue. According to the results of mercury measurements in various brain tissues, muscle, and blood, treadmill exercise itself did not influence mercury concentrations (Table 1). These results suggest that the inhibitory effect of treadmill exercise on neurological dysfunction is not caused by changes in tissue mercury concentrations.

## Conclusions

**Fig. 8:**
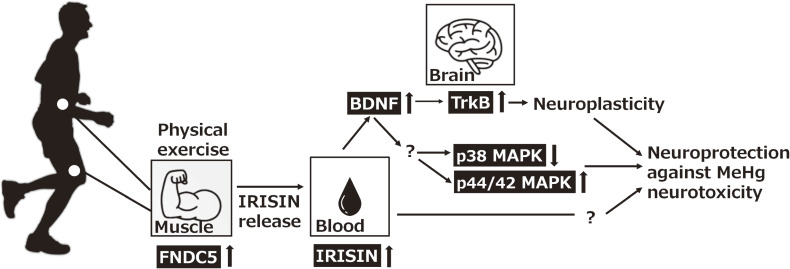
Possible protective mechanism of moderate exercise against MeHg neurotoxicity.

In summary, these results suggest that moderate exercise-induced increases in BDNF in the brain suppress MeHg neurotoxicity. Furthermore, there may be a mechanism by which exercise-induced increases in FNDC5 in muscle tissue are released into the blood as irisin, which is ultimately transported to the brain and promotes BDNF production (Fig. 8). Human exposure to MeHg contamination through gold mining remains a concern in lower-middle-income countries. The results from this study may contribute to establishing preventive medical measures against MeHg neurotoxicity.

## Abbreviations

MeHg: Methylmercury
BDNF: brain-derived neurotrophic factor
C57BL/6NJcl: C57 black 6NJcl
Trk: tropomyosin receptor kinase
MAPK: mitogen-activated protein kinase
FNDC5: fibronectin type III domain containing 5
NeuN: neuronal nuclei
Iba1: ionized calcium-binding adaptor molecule 1
GFAP: glial fibrillary acidic protein
TUNEL: deoxynucleotidyl transferase-mediated dUTP nick-end labeling
NTF: neurotrophic factor
NGF: nerve growth factor
NT: neurotrophin
SEM: standard error of the mean

## Declarations

### Availability of data and materials

The datasets generated and/or analyzed during the current study are available from the corresponding author upon reasonable request.

## References

[1] Kurland LT, Faro SN, Siedler H. Minamata disease. The outbreak of a neurologic disorder in Minamata, Japan, and its relationship to the ingestion of seafood contaminated by mercuric compounds. World Neurol. 1960;1:370–95. 13755288

[2] Kurland LT. Methyl-mercury sources, mode of action and clinical and pathological effects on the developing nervous system. Res Publ Assoc Res Nerv Ment Dis. 1973;51:283–97. 4590404

[3] Takizawa Y. Studies on the Niigata episode of Minamata disease outbreak. Investigation of causative agents of organic mercury poisoning in the district along the river Agano. Acta Med Biol (Niigata). 1970;17(4):293–7. 5464506

[4] Basu N, Bastiansz A, Dórea JG, Fujimura M, Horvat M, Shroff E, Weihe P, Zastenskaya I. Our evolved understanding of the human health risks of mercury. Ambio. 2023;52:877–96. doi: 10.1007/s13280-023-01831-6. 36790578 PMC10073381

[5] Eto K. Pathology of Minamata disease. Toxicol Pathol. 1997;25(6):614–23. doi: 10.1177/019262339702500612. 9437807

[6] Eto K, Takeuchi T. A pathological study of prolonged cases of Minamata disease. With particular reference to 83 autopsy cases. Acta Pathol Jpn. 1978;28(4):565–84. doi: 10.1111/j.1440-1827.1978.tb00896.x. 716883

[7] Eto K, Takizawa Y, Akagi H, Haraguchi K, Asano S, Takahata N, Tokunaga H. Differential diagnosis between organic and inorganic mercury poisoning in human cases-the pathologic point of view. Toxicol Pathol. 1999;27(6):664–71. doi: 10.1177/019262339902700608. 10588547

[8] Okajima T, Mishima I, Tokuomi H. Minamata disease with a long-term follow-up. Int J Neurol. 1976;11(1):62–72. 1017915

[9] Tokuomi H, Uchino M, Imamura S, Yamanaga H, Nakanishi R, Ideta T. Minamata disease (organic mercury poisoning): neuroradiologic and electrophysiologic studies. Neurology. 1982;32(12):1369–75. doi: 10.1212/WNL.32.12.1369. 6890643

[10] Schmidt-Kastner R, Freund TF. Selective vulnerability of the hippocampus in brain ischemia. Neuroscience. 1991;40(3):599–636. doi: 10.1016/0306-4522(91)90001-5. 1676492

[11] Haces ML, Montiel T, Massieu L. Selective vulnerability of brain regions to oxidative stress in a non-coma model of insulin-induced hypoglycemia. Neuroscience. 2010;165(1):28–38. doi: 10.1016/j.neuroscience.2009.10.003. 19818385

[12] Newell DW, Malouf AT, Franck JE. Glutamate-mediated selective vulnerability to ischemia is present in organotypic cultures of hippocampus. Neurosci Lett. 1990;116(3):325–30. doi: 10.1016/0304-3940(90)90095-Q. 1978744

[13] Vornov JJ, Tasker RC, Coyle JT. Direct observation of the agonist-specific regional vulnerability to glutamate, NMDA, and kainite neurotoxicity in organotypic hippocampal cultures. Exp Neurol. 1991;114(1):11–22. doi: 10.1016/0014-4886(91)90079-R. 1717307

[14] Wilde GJ, Pringle AK, Wright P, Iannotti F. Differential vulnerability of the CA1 and CA3 subfields of the hippocampus to superoxide and hydroxyl radicals *in vitro*. J Neurochem. 1997;69(2):883–6. doi: 10.1046/j.1471-4159.1997.69020883.x. 9231752

[15] Fujimura M, Usuki F, Sawada M, Takashima A. Methylmercury induces neuropathological changes with tau hyperphosphorylation mainly through the activation of the c-jun-N-terminal kinase pathway in the cerebral cortex, but not in the hippocampus of the mouse brain. Neurotoxicology. 2009;30(6):1000–7. doi: 10.1016/j.neuro.2009.08.001. 19666049

[16] Fujimura M, Usuki F, Nakamura A. Methylmercury induces hyperalgesia/allodynia through spinal cord dorsal horn neuronal activation and subsequent somatosensory cortical circuit formation in rats. Arch Toxicol. 2021;95:2151–62. doi: 10.1007/s00204-021-03047-7. 33847776

[17] Fujimura M, Unoki T. Preliminary evaluation of the mechanism underlying vulnerability/resistance to methylmercury toxicity by comparative gene expression profiling of rat primary cultured cerebrocortical and hippocampal neurons. J Toxicol Sci. 2022;47(5):211–9. doi: 10.2131/jts.47.211. 35527009

[18] Fujimura M, Unoki T. BDNF specifically expressed in hippocampal neurons is involved in methylmercury neurotoxicity resistance. Environ Toxicol. 2024;39(5):3149–59. doi: 10.1002/tox.24174. 38323385

[19] Park H, Poo MM. Neurotrophin regulation of neural circuit development and function. Nat Rev Neurosci. 2013;14:7–23. doi: 10.1038/nrn3379. 23254191

[20] Minichiello L. TrkB signalling pathways in LTP and learning. Nat Rev Neurosci. 2009;10:850–60. doi: 10.1038/nrn2738. 19927149

[21] Neeper SA, Gómez-Pinilla F, Choi J, Cotman CW. Exercise and brain neurotrophins. Nature. 1995;373:109. doi: 10.1038/373109a0. 7816089

[22] Neeper SA, Gómez-Pinilla F, Choi J, Cotman CW. Physical activity increases mRNA for brain-derived neurotrophic factor and nerve growth factor in rat brain. Brain Res. 1996;726(1–2):49–56. doi: 10.1016/0006-8993(96)00273-9. 8836544

[23] Gómez-Pinilla F, Ying Z, Opazo P, Roy RR, Edgerton VR. Differential regulation by exercise of BDNF and NT-3 in rat spinal cord and skeletal muscle. Eur J Neurosci. 2001;13(6):1078–84. doi: 10.1046/j.0953-816x.2001.01484.x. 11285004

[24] Adlard PA, Perreau VM, Engesser-Cesar C, Cotman CW. The timecourse of induction of brain-derived neurotrophic factor mRNA and protein in the rat hippocampus following voluntary exercise. Neurosci Lett. 2004;363(1):43–8. doi: 10.1016/j.neulet.2004.03.058. 15157993

[25] Inoue T, Ninuma S, Hayashi M, Okuda A, Asaka T, Maejima H. Effects of long-term exercise and low-level inhibition of GABAergic synapses on motor control and the expression of BDNF in the motor related cortex. Neurol Res. 2018;40(1):18–25. doi: 10.1080/01616412.2017.1382801. 29019708

[26] Gómez-Pinilla F, Ying Z, Roy RR, Molteni R, Edgerton VR. Voluntary exercise induces a BDNF-mediated mechanism that promotes neuroplasticity. J Neurophysiol. 2002;88(5):2187–95. doi: 10.1152/jn.00152.2002. 12424260

[27] Gómez-Pinilla F, Zhuang Y, Feng J, Ying Z, Fan G. Exercise impacts brain-derived neurotrophic factor plasticity by engaging mechanisms of epigenetic regulation. Eur J Neurosci. 2011;33(3):383–90. doi: 10.1111/j.1460-9568.2010.07508.x. 21198979 PMC3256007

[28] Maejima H, Ninuma S, Okuda A, Inoue T, Hayashi M. Exercise and low-level GABA_A_ receptor inhibition modulate locomotor activity and the expression of BDNF accompanied by changes in epigenetic regulation in the hippocampus. Neurosci Lett. 2018;685:18–23. doi: 10.1016/j.neulet.2018.07.009. 30037768

[29] Cefis M, Prigent-Tessier A, Quirié A, Pernet N, Marie C, Garnier P. The effect of exercise on memory and BDNF signaling is dependent on intensity. Brain Struct Funct. 2019;224:1975–85. doi: 10.1007/s00429-019-01889-7. 31089854

[30] Rasmussen P, Brassard P, Adser H, Pedersen MV, Leick L, Hart E, Secher NH, Pedersen BK, Pilegaard H. Evidence for a release of brain-derived neurotrophic factor from the brain during exercise. Exp Physiol. 2009;94(10):1062–9. doi: 10.1113/expphysiol.2009.048512. 19666694

[31] Zafra F, Hengerer B, Leibrock J, Thoenen H, Lindholm D. Activity dependent regulation of BDNF and NGF mRNAs in the rat hippocampus is mediated by non-NMDA glutamate receptors. EMBO J. 1990;9:3545–50. doi: 10.1002/j.1460-2075.1990.tb07564.x. 2170117 PMC552104

[32] Chen K, Zheng Y, Wei JA, Ouyang H, Huang X, Zhang F, Lai CSW, Ren C, So KF, Zhang L. Exercise training improves motor skill learning via selective activation of mTOR. Sci Adv. 2019;5(7):eaaw1888. doi: 10.1126/sciadv.aaw1888. 31281888 PMC6609215

[33] Isaac AR, Lima-Filho RAS, Lourenco MV. How does the skeletal muscle communicate with the brain in health and disease? Neuropharmacology. 2021;197:108744. doi: 10.1016/j.neuropharm.2021.108744. 34363812

[34] Pedersen BK. Physical activity and muscle-brain crosstalk. Nat Rev Endocrinol. 2019;15:383–92. doi: 10.1038/s41574-019-0174-x. 30837717

[35] Horowitz AM, Fan X, Bieri G, Smith LK, Sanchez-Diaz CI, Schroer AB, Gontier G, Casaletto KB, Kramer JH, Williams KE, Villeda SA. Blood factors transfer beneficial effects of exercise on neurogenesis and cognition to the aged brain. Science. 2020;369(6500):167–73. doi: 10.1126/science.aaw2622. 32646997 PMC7879650

[36] De Miguel Z, Khoury N, Betley MJ, Lehallier B, Willoughby D, Olsson N, Yang AC, Hahn O, Lu N, Vest RT, Bonanno LN, Yerra L, Zhang L, Saw NL, Fairchild JK, Lee D, Zhang H, McAlpine PL, Contrepois K, Shamloo M, Elias JE, Rando TA, Wyss-Coray T. Exercise plasma boosts memory and dampens brain inflammation via clusterin. Nature. 2021;600:494–9. doi: 10.1038/s41586-021-04183-x. 34880498 PMC9721468

[37] Fujimura M. Gabapentin improves neuropathic pain in Minamata disease model rats. Environ Health Prev Med. 2024;29:31. doi: 10.1265/ehpm.24-00035. 38825526 PMC11157338

[38] Fujimura M, Usuki F, Unoki T. Decreased plasma thiol antioxidant capacity precedes neurological signs in a rat methylmercury intoxication model. Food Chem Toxicol. 2020;146:111810. doi: 10.1016/j.fct.2020.111810. 33058990

[39] Misu H, Takayama H, Saito Y, Mita Y, Kikuchi A, Ishii KA, Chikamoto K, Kanamori T, Tajima N, Lan F, Takeshita Y, Honda M, Tanaka M, Kato S, Matsuyama N, Yoshioka Y, Iwayama K, Tokuyama K, Akazawa N, Maeda S, Takekoshi K, Matsugo S, Noguchi N, Kaneko S, Takamura T. Deficiency of the hepatokine selenoprotein P increases responsiveness to exercise in mice through upregulation of reactive oxygen species and AMP-activated protein kinase in muscle. Nat Med. 2017;23:508–16. doi: 10.1038/nm.4295. 28263310

[40] Li Q, Ishii KA, Kamoshita K, Takahashi K, Abuduwaili H, Takayama H, Galicia-Medina CM, Tanida R, Ko Oo H, Gafiyatullina G, Yao X, Abuduyimiti T, Hamazaki J, Goto H, Nakano Y, Takeshita Y, Harada K, Murata S, Takamura T. PAC1 deficiency protects obese male mice from immobilization-induced muscle atrophy by suppressing FoxO-Atrogene axis. Endocrinology. 2023;164(6):bqad065. doi: 10.1210/endocr/bqad065. 37103220 PMC10205472

[41] Um HS, Kang EB, Koo JH, Kim HT, Jin-Lee, Kim EJ, Yang CH, An GY, Cho IH, Cho JY. Treadmill exercise represses neuronal cell death in an aged transgenic mouse model of Alzheimer’s disease. Neurosci Res. 2011;69(2):161–73. doi: 10.1016/j.neures.2010.10.004. 20969897

[42] Koo JH, Kwon IS, Kang EB, Lee CK, Lee NH, Kwon MG, Cho IH, Cho JY. Neuroprotective effects of treadmill exercise on BDNF and P13-K/Akt signaling pathway in the cortex of transgenic mice model of Alzheimer’s disease. J Exerc Nutrition Biochem. 2013;17(4):151–60. doi: 10.5717/jenb.2013.17.4.151. PMC4241914 25566426

[43] Randazzo D, Blaauw B, Paolini C, Pierantozzi E, Spinozzi S, Lange S, Chen J, Protasi F, Reggiani C, Sorrentino V. Exercise-induced alterations and loss of sarcomeric M-line organization in the diaphragm muscle of obscurin knockout mice. Am J Physiol Cell Physiol. 2016;312(1):C16–28. doi: 10.1152/ajpcell.00098.2016. 27784675 PMC5283896

[44] Yoshikawa A, Ohtaki H, Miyamoto K, Kim S, Hase K, Yoshida M, Kamijo S, Kamimura S, Koiwa N, Izumizaki M. Mild-intensity running exercise recovered motor function by improvement of ankle mobility after unilateral brain injury of mice using three-dimensional kinematic analysis techniques. Brain Res. 2023;1798:148160. doi: 10.1016/j.brainres.2022.148160. 36372237

[45] Nomura R, Takasugi N, Hiraoka H, Iijima Y, Iwawaki T, Kumagai Y, Fujimura M, Uehara T. Alterations in UPR signaling by methylmercury trigger neuronal cell death in the mouse brain. Int J Mol Sci. 2022;23(23):15412. doi: 10.3390/ijms232315412. 36499738 PMC9738736

[46] Iijima Y, Miki R, Fujimura M, Oyadomari S, Uehara T. Methylmercury-induced brain neuronal death in CHOP-knockout mice. J Toxicol Sci. 2024;49(2):55–60. doi: 10.2131/jts.49.55. 38296529

[47] Miki R, Nomura R, Iijima Y, Kubota S, Takasugi N, Iwawaki T, Fujimura M, Uehara T. Therapeutic potential of 4-phenylbutyric acid against methylmercury-induced neuronal cell death in mice. Arch Toxicol. 2025;99:563–74. doi: 10.1007/s00204-024-03902-3. 39465421 PMC11775073

[48] Fujimura M, Usuki F. Pregnant rats exposed to low-level methylmercury exhibit cerebellar synaptic and neuritic remodeling during the perinatal period. Arch Toxicol. 2020;94:1335–47. doi: 10.1007/s00204-020-02696-4. 32140736

[49] Jeneson JA, de Snoo MW, Verlinden NA, Joosten BJ, Doornenbal A, Schot A, Everts ME. Treadmill but not wheel running improves fatigue resistance of isolated extensor digitorum longus muscle in mice. Acta Physiol (Oxf). 2007;190(2):151–61. doi: 10.1111/j.1748-1716.2007.01680.x. 17394571

[50] Fujimura M, Cheng J, Zhao W. Perinatal exposure to low-dose methylmercury induces dysfunction of motor coordination with decreases in synaptophysin expression in the cerebellar granule cells of rats. Brain Res. 2012;1464:1–7. doi: 10.1016/j.brainres.2012.05.012. 22587888

[51] Fujimura M, Usuki F, Nakamura A. Fasudil, a rho-associated coiled coil-forming protein kinase inhibitor, recovers methylmercury-induced axonal degeneration by changing microglial phenotype in rats. Toxicol Sci. 2019;168(1):126–36. doi: 10.1093/toxsci/kfy281. 30462329

[52] Fujimura M, Usuki F. Methylmercury induces oxidative stress and subsequent neural hyperactivity leading to cell death through the p38 MAPK-CREB pathway in differentiated SH-SY5Y cells. Neurotoxicology. 2018;67:226–33. doi: 10.1016/j.neuro.2018.06.008. 29913201

[53] Warburton DER, Bredin SSD. Health benefits of physical activity: a systematic review of current systematic reviews. Curr Opin Cardiol. 2017;32(5):541–56. doi: 10.1097/HCO.0000000000000437. 28708630

[54] Cabral DF, Rice J, Morris TP, Rundek T, Pascual-Leone A, Gomes-Osman J. Exercise for brain health: an investigation into the underlying mechanisms guided by dose. Neurotherapeutics. 2019;16(3):580–99. doi: 10.1007/s13311-019-00749-w. 31197642 PMC6694330

[55] Grasgruber P, Hrazdíra E. Nutritional and socio-economic predictors of adult height in 152 world populations. Econ Hum Biol. 2020;37:100848. doi: 10.1016/j.ehb.2020.100848. 32247188

[56] Fujimura M, Usuki F. Site-specific neural hyperactivity via the activation of MAPK and PKA/CREB pathways triggers neuronal degeneration in methylmercury-intoxicated mice. Toxicol Lett. 2017;271:66–73. doi: 10.1016/j.toxlet.2017.03.001. 28267559

[57] Fujimura M. Fasudil, a ROCK inhibitor, prevents neuropathic pain in Minamata disease model rats. Toxicol Lett. 2022;371:38–45. doi: 10.1016/j.toxlet.2022.10.001. 36244566

[58] Fujimura M, Usuki F. Cellular conditions responsible for methylmercury-mediated neurotoxicity. Int J Mol Sci. 2022;23(13):7218. doi: 10.3390/ijms23137218. 35806222 PMC9266708

[59] Vakili Zahir N, Abkhezr M, Khaje Piri Z, Ostad SN, Kebriaezade A, Ghahremani MH. The time course of JNK and P38 activation in cerebellar granule neurons following glucose deprivation and BDNF treatment. Iran J Pharm Res. 2012;11(1):315–23. 24250454 PMC3813105

[60] Sarker KP, Biswas KK, Rosales JL, Yamaji K, Hashiguchi T, Lee KY, Maruyama I. Ebselen inhibits NO-induced apoptosis of differentiated PC12 cells via inhibition of ASK1-p38 MAPK-p53 and JNK signaling and activation of p44/42 MAPK and Bcl-2. J Neurochem. 2003;87(6):1345–53. doi: 10.1046/j.1471-4159.2003.02096.x. 14713291

[61] Yeo JE, Kang SK. Selenium effectively inhibits ROS-mediated apoptotic neural precursor cell death in vitro and in vivo in traumatic brain injury. Biochim Biophys Acta. 2007;1772(11–12):1199–210. doi: 10.1016/j.bbadis.2007.09.004. 17997286

[62] Kamada Y, Toyama S, Arai Y, Inoue H, Nakagawa S, Fujii Y, Kaihara K, Kishida T, Mazda O, Takahashi K. Treadmill running prevents atrophy differently in fast- versus slow-twitch muscles in a rat model of rheumatoid arthritis. Muscle Res Cell Motil. 2021;42:429–41. doi: 10.1007/s10974-021-09610-0. 34687403

[63] Wen P, Sun Z, Yang D, Li J, Li Z, Zhao M, Wang D, Gou F, Wang J, Dai Y, Zhao D, Yang L. Irisin regulates oxidative stress and mitochondrial dysfunction through the UCP2-AMPK pathway in prion diseases. Cell Death Dis. 2025;16:66. doi: 10.1038/s41419-025-07390-w. 39900919 PMC11790890

[64] Choi JW, Jo SW, Kim DE, Paik IY, Balakrishnan R. Aerobic exercise attenuates LPS-induced cognitive dysfunction by reducing oxidative stress, glial activation, and neuroinflammation. Redox Biol. 2024;71:103101. doi: 10.1016/j.redox.2024.103101. 38408409 PMC10904279

[65] Liu Y, Liu Y, Zhang X, Yan G, Qi L, Yong VW, Xue M. The cerebroprotection and prospects of FNDC5/irisin in stroke. Neuropharmacology. 2024;253:109986. doi: 10.1016/j.neuropharm.2024.109986. 38705569

[66] Li N, Wang B, Wang Y, Tian X, Lin J, Sun X, Sun Y, Zhang X, Xu H, Li M, Zeng F, Zhao R. Exercise Ameliorates Dysregulated Mitochondrial Fission, Mitochondrial Respiration, and Neuronal Apoptosis in Parkinson’s Disease Mice via the Irisin/AMPK/SIRT1 Pathway. Mol Neurobiol. 2025;62:8843–56. doi: 10.1007/s12035-025-04801-z. 40048058

